# Step-by-step bailout for MitraClip lock line stuck due to a knot

**DOI:** 10.1093/ehjcr/ytaf510

**Published:** 2025-10-08

**Authors:** Yuta Kobayashi, Yusuke Enta, Yoshiko Munehisa, Norio Tada

**Affiliations:** Department of Cardiology, Sendai Kousei Hospital, 1-20, Tsutsumidori Amamiya-cho, Aoba-ku, Sendai, Miyagi 981-0914, Japan; Department of Cardiovascular Medicine, Hokkaido University Hospital, Kita 15, Nishi 7, Kita-ku, Sapporo, Hokkaido 060-8638, Japan; Department of Cardiology, Sendai Kousei Hospital, 1-20, Tsutsumidori Amamiya-cho, Aoba-ku, Sendai, Miyagi 981-0914, Japan; Department of Laboratory Medicine, The Jikei University School of Medicine, 3-19-18 Nishi-Shimbashi, Minato Ward, Tokyo 105-0003, Japan; Department of Cardiology, Sendai Kousei Hospital, 1-20, Tsutsumidori Amamiya-cho, Aoba-ku, Sendai, Miyagi 981-0914, Japan; Department of Cardiology, Sendai Kousei Hospital, 1-20, Tsutsumidori Amamiya-cho, Aoba-ku, Sendai, Miyagi 981-0914, Japan

**Keywords:** M-TEER, Mitral regurgitation

## Summary

An 84-year-old man with prior transcatheter edge-to-edge repair for P2 prolapse presented with recurrent primary mitral regurgitation (pMR). A second MitraClip NTW was placed adjacent to the prior implant, but during deployment, the lock line became immobile after partial withdrawal. The clip could not be reopened, and one end of the lock line had already entered the delivery catheter handle. We proceeded to retrieve the clip and clip delivery system (CDS) together. Coaxial alignment among the steerable guide catheter (SGC), CDS, and the clip was achieved by adjusting the M and + Knobs, enabling smooth removal. A lock line knot was confirmed after device retrieval. This case highlights the importance of inspecting the lock line before withdrawal and demonstrates a safe bailout technique when entrapment occurs.

## Case description

We report the case of an 84-year-old man with prior transcatheter edge-to-edge mitral valve repair (M-TEER) for pMR caused by P2 prolapse. He developed recurrent pMR, leading to heart failure unresponsive to medical therapy. At the time of presentation, his medications included furosemide 40 mg daily and sacubitril/valsartan 400 mg daily. Given the persistence of symptoms, reintervention with the MitraClip system was undertaken.

A MitraClip NTW was placed at the A2–P2, adjacent to a previous implant, effectively reducing mitral regurgitation (MR). However, during deployment, the lock line became stuck after approximately 10 cm had been pulled. One end had already retracted into the delivery catheter handle, making retrieval from the opposite side impossible. Attempts to reopen and retrieve the clip failed and it was considered irretrievable.

**Figure 1:**
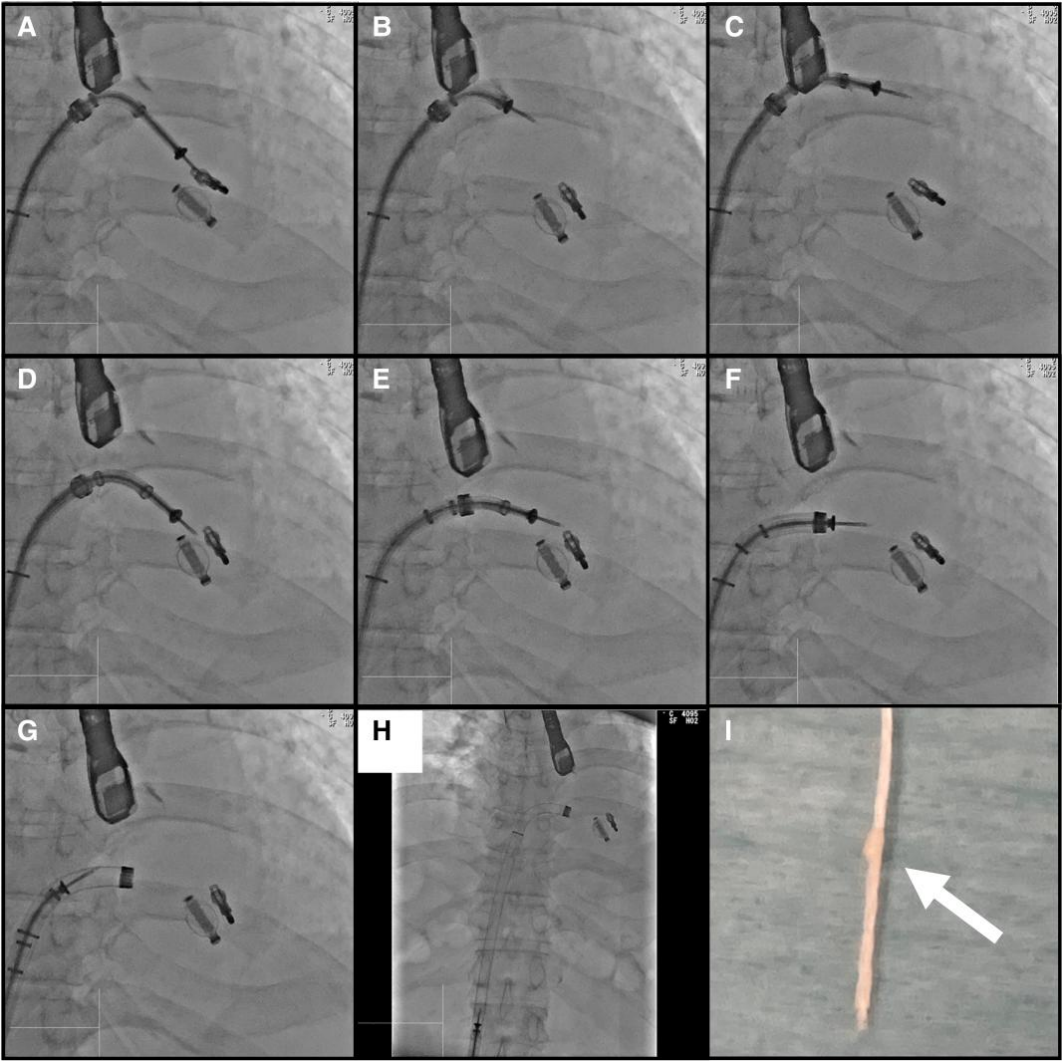
Fluoroscopic image and removed lock line. (*A–C*) Clip tilts after M knob release. (*D–H*) M and + knobs adjustments restore coaxial alignment, allowing clip delivery system removal. (*I*) Knot in the lock line.

We proceeded to remove the CDS with the clip and lock line still attached. To enable retraction into the SGC, the M knob was released to shift the CDS away from the valve. However, this caused the clip to tilt due to off-axis tension (*Figures 1A–C*, Supplementary material online, *Video S1*). To correct this, the M and + knobs were adjusted, bringing the SGC closer to the clip and restoring coaxial alignment under fluoroscopic guidance (*Figures 1D–H*, Supplementary material online, *Videos S2* and *S3*). This allowed safe retraction of the CDS.

The CDS was safely removed without complication, and a knot was detected in the lock line (*Figure 1I*). The procedure was completed without MR deterioration or valve injury. Afterward, his medical regimen was adjusted to furosemide 20 mg daily, while sacubitril/valsartan 400 mg daily was continued. At 1-year follow-up, echocardiography showed no recurrence of MR and no evidence of valve injury.

Lock line stuck may occur during clip deployment. If this occurs, retrieval of the clip should be attempted. However, if retrieval fails, advancing the SGC and maintaining a coaxial alignment under fluoroscopic guidance allows safe CDS removal. To prevent such complications, it is essential to confirm the absence of knots before withdrawal and to pull the lock line in a coaxial direction.

## Supplementary Material

ytaf510_Supplementary_Data

## Data Availability

All data related to this case report are presented in the published manuscript.

## Notes

**Statement of consent:** The authors confirm that written consent for submission and publication of this case report including images and associated texts has been obtained from all patients in line with COPE guidance.

**Funding:** None declared.

